# Long-term survival, radiological, and histomorphological evaluation of hip resurfacing arthroplasty (HemiCAP) in the treatment of femoral head osteonecrosis: An 11-year follow-up case report

**DOI:** 10.1016/j.ijscr.2025.112095

**Published:** 2025-10-24

**Authors:** Onur Bilge, Muhammed Furkan Küçükşen, Mustafa Cihat Avunduk, Ülkü Kerimoğlu

**Affiliations:** a Necmettin Erbakan University, Meram Faculty of Medicine, Department of Orthopedics and Traumatology, Konya, Turkey; b Necmettin Erbakan University, Meram Faculty of Medicine, Department of Sports Medicine, Konya, Turkey; c Necmettin Erbakan University, Meram Medicine Faculty, Department of Pathology, Konya, Turkey; d Necmettin Erbakan University, Meram Medicine Faculty, Department of Radyology, Konya, Turkey

**Keywords:** HemiCAP, Hip resurfacing arthroplasty, Femoral head osteonecrosis, Osseointegration, Histomorphological evaluation, Case report

## Abstract

**Introduction and importance:**

To the best of our knowledge, the histomorphological and radiological evaluation and long-term survival of osseointegration of the HemiCAP® focal hip resurfacing system in the treatment of femoral head osteonecrosis have not previously been demonstrated. This study presents a case of a patient with femoral head osteonecrosis who underwent HemiCAP® resurfacing arthroplasty, followed by total hip arthroplasty (THA) after 11 years. Radiological and histomorphological assessments were performed on the removed implant. A review of relevant literature is also included.

**Case presentation:**

A 54-year-old female with right femoral head osteonecrosis did not respond to three years of conservative treatment and core decompression. In 2013, she underwent HemiCAP® resurfacing. No complications such as subsidence, periprosthetic fracture, femoral neck narrowing, osteolysis, or prosthetic infection were observed over the 11-year follow-up. In 2024, THA was performed due to osteoarthritis progression. The explanted femoral head and implant were analyzed histomorphologically and radiologically.

**Clinical discussion:**

This case demonstrates that the HemiCAP® implant achieved uneventful osseointegration over 11 years. These findings, supported by long-term radiological and histomorphometric evaluation, highlight the implant's potential value in joint-preserving surgeries in terms of long-term survival.

**Conclusion:**

This case demonstrates that HemiCAP® resurfacing can provide 11-year implant survival without failure. To our knowledge, this is the first report evaluating both radiological and histological osseointegration of a HemiCAP® hip implant after long-term follow-up.

## Highlights

• First long term histomorphological analysis of hip HemiCAP
• HemiCAP provided 11-year survival in femoral head osteonecrosis.
• No signs of implant failure were observed over a decade of follow-up.
• Implant osseointegration evaluated after conversion to total hip arthroplasty.
• Adds rare long-term outcome data to focal hip resurfacing literature

## 1 Introduction

Femoral head osteonecrosis is a painful, progressive, and potentially disabling disease with various etiologies, including trauma, steroid use, and alcohol consumption. The management of osteonecrosis should begin with a high level of suspicion to enable early diagnosis before collapse occurs and to prevent osteoarthritis [1]. Several joint-preserving treatment options, such as resurfacing arthroplasties and core decompression, are employed to delay disease progression. However, total joint replacement remains the definitive treatment option for osteonecrosis.

In recent years, various modalities have been proposed for managing osteonecrosis with joint-preserving surgical techniques. However, current literature provides limited evidence regarding the long-term effectiveness of these techniques [2]. There is limited evidence on the long-term survival and biological effects of HemiCAP® resurfacing arthroplasty, particularly regarding bone remodeling and stress shielding. Although it may reduce bone loss, definitive data on its compatibility with long-term durability are lacking. Current literature underscores the need for large-scale, comprehensive studies on long-term outcomes [[1], [2], [3]]. The current literature highlights a lack of comprehensive and large-scale research on the long-term outcomes of this method.

Osseointegration refers to the direct and stable connection between implants and living bone tissue, playing a crucial role in the long-term success of implants [4]. The long-term success of orthopedic implants is directly related to osseointegration, which is influenced by both the surrounding bone tissue and the implant surface [5]. Research has shown that surface characteristics, biomechanical compatibility, and surgical techniques significantly impact the osseointegration process and implant success [6].

To the best of our knowledge, there has been no previously reported case of a joint-preserving, anatomically focused resurfacing implant (HemiCAP®) with a long survival period of eleven years, in which the histomorphometric osseointegration and bone integration of the implant were evaluated.

This case has been reported in accordance with the updated SCARE 2025 criteria [7]. nd the PROCESS 2025 guidelines [8].

## 2 Case presentation

A 54-year-old female patient, diagnosed with avascular necrosis of the right femoral head in 2010, was treated non-surgically for two years with nonsteroidal anti-inflammatory drugs (NSAIDs) and crutch-assisted weight-bearing reduction. In 2012, she presented to our adult reconstructive surgery clinic with complaints of right hip pain and restricted joint motion. She had no history of trauma. The pain was localized to the right groin and worsened with joint movements, particularly during internal rotation and weight-bearing. That year, core decompression was performed; however, both surgical and conservative treatments provided only limited symptom relief. Due to persistent complaints, the patient underwent HemiCAP® resurfacing arthroplasty in 2013.

## 3 Operative and postoperative periods

**Fig. 1:**
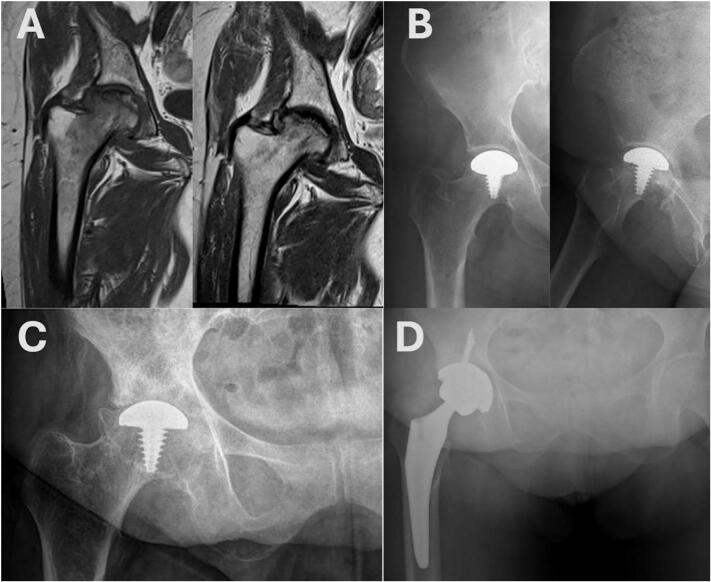
T1-weighted MRI sequences of the right hip obtained, respectively, prior to core decompression and immediately before HemiCAP® resurfacing arthroplasty (drill holes in the femoral neck from the core decompression procedure can be observed). (A) Anteroposterior and lateral hip radiographs of the patient (respectively) at postoperative year 1 following HemiCAP® resurfacing arthroplasty. (B). Preoperative anteroposterior pelvic radiograph of the patient prior to total hip arthroplasty (C). Anteroposterior pelvic radiograph of the patient at postoperative year 1 following HemiCAP® resurfacing arthroplasty (D).

Under spinal anesthesia, a safe surgical dislocation was performed using the Ganz technique. The osteonecrotic area was debrided, and a conical screw was placed. A 35 mm HemiCAP® (Arthrosurface, Franklin, MA) implant was securely fixed onto the screw, positioned 0.5 mm below the level of the surrounding healthy cartilage [9]. The hip was relocated, range of motion was assessed, and the osteotomy was closed. No complications occurred. Thromboprophylaxis was administered for four weeks. The patient was mobilized with gradual progression to full weight-bearing to facilitate osteotomy healing and implant integration. At the postoperative one-year follow-up following HemiCAP® resurfacing arthroplasty, complete union of the osteotomy site was observed, and the clinical and radiographic outcomes were satisfactory (Fig. 1B).

During 11-year follow-up period, no signs of failure such as subsidence, periprosthetic fracture, femoral neck narrowing, osteolysis, or prosthetic infection were observed. After this prolonged long term follow-up period the patient underwent revision to THA in 2024 due to the progression of osteoarthritis (Fig. 1C, D). The revision utilized a Trident PSL HA acetabular system (52-mm shell, 10° polyethylene liner, fixed with one 6.5 × 16 mm and two 6.5 × 35 mm screws) and an Accolade II femoral stem (size 6, 132° neck) with a 36-mm BIOLOX Delta ceramic head (0-mm offset) (Howmedica Osteonics, Mahwah, NJ, USA) (Fig. 1D).

**Fig. 2:**
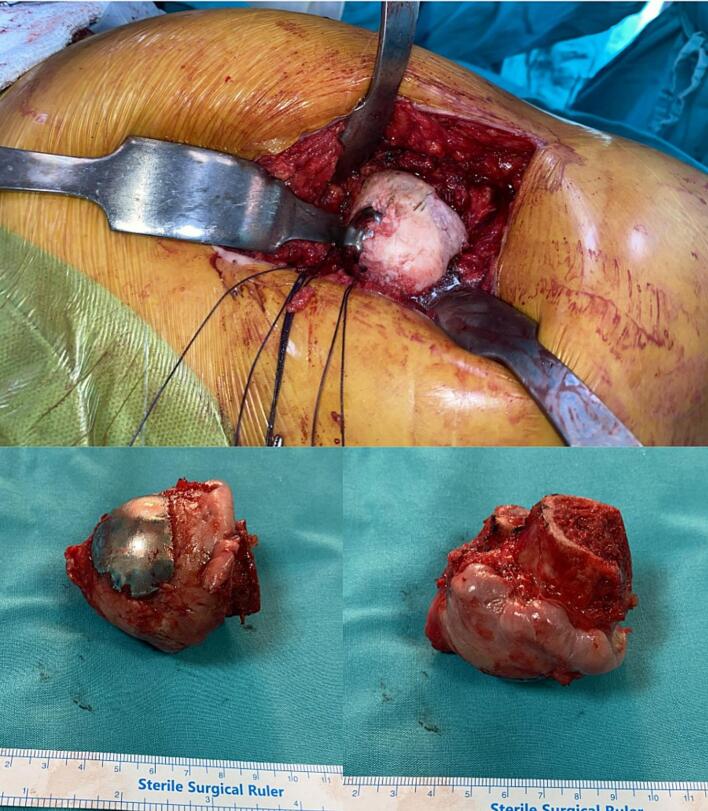
Intraoperative images showing a stably fixed resurfaced femoral head with preserved femoral neck. Note the cartilage loss, degenerative changes, and loss of spherical contour.

During surgery, the resurfaced femoral head was found to be stably fixed, and the femoral neck was preserved. There was marked cartilage loss and surface irregularities of the femoral head with degenerative changes of osteoarthritis, and its spherical morphology was compromised (Fig. 2). The femoral head, along with the removed HemiCap prosthesis, was examined radiologically and histomorphologically.

## 4 Radiological evaluation

**Fig. 3:**
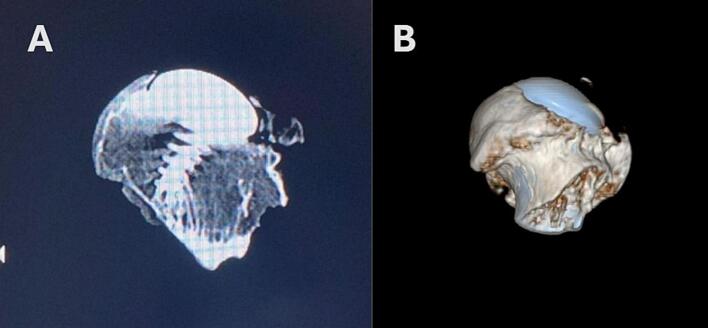
Coronal (A) and volume rendering reformatted (B) CT images demonstrate the integration of resurfacing impant with the bone and the absence of loosening or fracture.

At the end of 11 years, no failures or radiographic findings were observed related to subsidence, periprosthetic fracture, neck narrowing, or osteolysis. The anteroposterior (AP) and lateral pelvic X-ray taken 11.5 years postoperatively shows complete loss of the joint space (Fig. 1C). The microradiograph indicates that the trabecular structure of the bone has been preserved. After removal of femur, CT scan and volume rendering reconstructed images were obtained (Siemens Healthineers, Erlangen, Germany) (Fig. 3).

## 5 Histomorphological evaluation

### 5.1 Macroscopic evaluation (Fig. 4)

The trabecular bone surrounding the HemiCAP® implant was macroscopically preserved, showing direct bone–implant contact without interfacial gaps, indicating strong biomechanical integration and biocompatibility. Stable integration was noted at the head–neck junction, with minimal femoral neck bone loss. Bone tissue viability was largely maintained, with new bone formation around necrotic areas and signs of ongoing regeneration and remodeling, despite limited osteopenia.

**Fig. 4:**
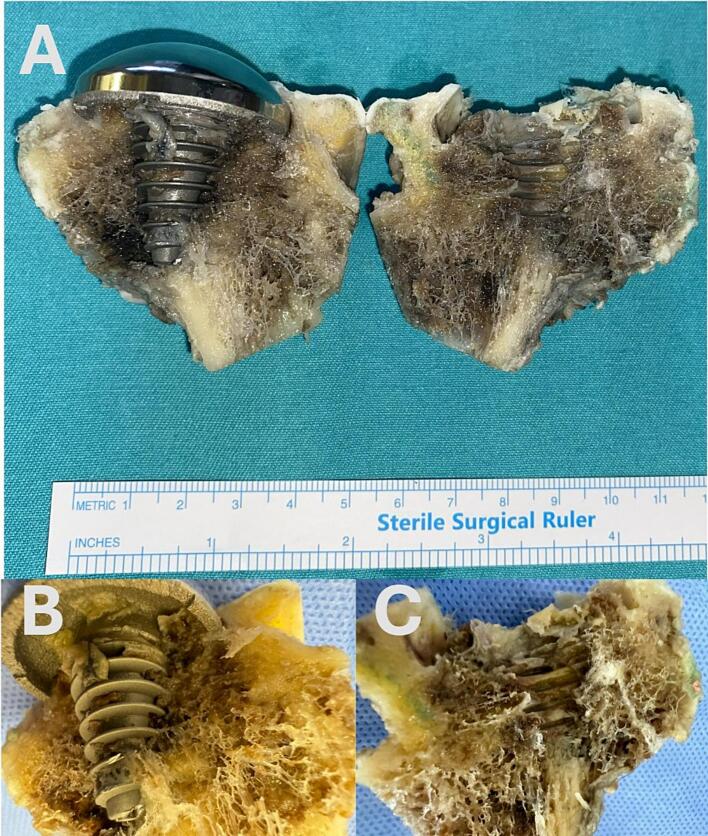
The bone embedded screw and the bone tissue around the screw are seen. The compatibility of the screw with the tissue is remarkable.

### 5.2 High-magnification imaging by SMZ microscope (Fig. 5)

When the lesion area was examined under high magnification using a stereo zoom (SMZ) microscope, the interface between the HemiCAP® resurfacing arthroplasty implant and the surrounding tissue was observed more distinctly. The morphological integrity and viability of the peri-implant tissue could be clearly distinguished, indicating that the tissue maintained structural and functional integration with the implant.

**Fig. 5:**
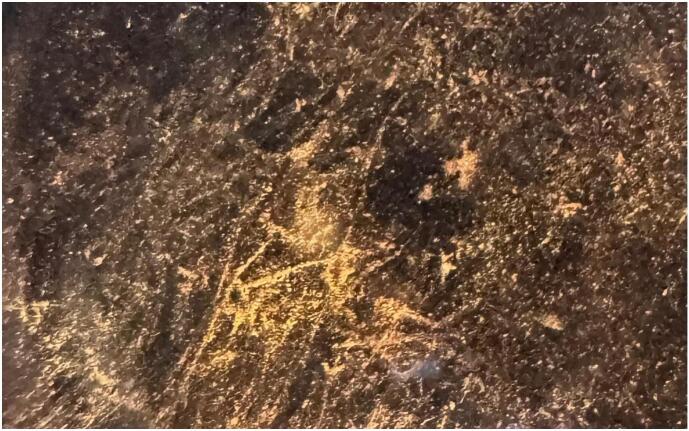
When the lesion area is examined with a SMZ (stereo zoom microscope) microscope at high magnification, the screw-tissue compatibility is seen more clearly. The vitality of the surrounding tissue is selected.

### 5.3 Histological evaluation by light microscopy (Fig. 6)

Decalcified bone sections were stained with hematoxylin–eosin (H&E) for morphology and von Gieson for connective tissue. As the femoral head specimen was decalcified to enable high-quality sectioning and immunohistochemistry, the gold standard Von Kossa technique could not be applied. Since decalcification removes inorganic calcium, H&E and von Gieson were used to assess the bone–implant interface and trabecular organization. Light microscopy demonstrated connective tissue adjacent to the HemiCAP® implant, surrounded by stromal tissue and mature organized bone. This architecture indicates progressive peri-implant remodeling and supports implant stability.

**Fig. 6:**
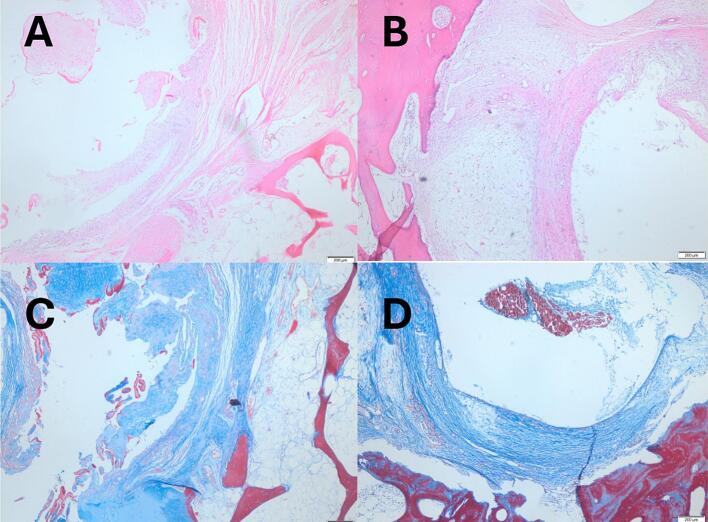
Light microscopic imaging of the area around the screw (A, B: HE, C, D: von Gieson). Connective tissue is seen immediately around the screw, supporting tissue is seen around the connective tissue, and bone tissue is seen around these.

## 6 Discussion

To the best of our knowledge, this study is the first report presenting not only the long-term radiological outcomes of focal anatomical surface resurfacing implantation treatment in a patient diagnosed with femoral head avascular necrosis but also the histomorphometric osteointegration of the prosthesis and peri-implant bone assessment.

In many studies, the osseointegration of implants, the presence of peri-implant osteolysis, and the longevity of implants have been evaluated histomorphometrically in addition to radiological and clinical assessments [10]. Furthermore, numerous studies, including those on hemisurface arthroplasties, have evaluated arthroplasty implants both radiologically and histomorphometrically [5,11]. Doria et al. conducted an experimental study on sheep to evaluate hip hemiarthroplasty using a hydroxyapatite-coated, specially designed femoral component, assessing radiological outcomes and prosthetic osseointegration via scanning electron microscopy [12]. Coathup et al. analyzed autopsy samples from patients with bimetric hip hemiarthroplasty using image analysis and morphometry, demonstrating that hydroxyapatite coating enhanced bone ingrowth and attachment, promoted uniform bone distribution, reduced stress shielding and wear particle-induced osteolysis, thereby supporting implant longevity and function [6].

To our knowledge, the number of case series in the literature on HemiCAP® hip surface arthroplasty is highly limited, with the longest follow-up duration reported being six years [3]. Although HemiCAP® implants offer the advantage of preserving the anatomical structure of the joint surface with minimal bone resection, a significant decline in their usage has been observed. This is due to complications such as implant failure, progressive arthritis, or fractures, which, despite delaying the need for total joint arthroplasty, do not entirely eliminate it [13]. However, the decline in HemiCAP® usage in current practice may also be associated with insufficient research on its long-term outcomes. Therefore, further studies are needed to determine the role and long-term effectiveness of HemiCAP® prostheses in joint-preserving surgeries.

Based on these findings, this study holds significance in the literature as the first to assess the osteointegration of HemiCAP® surface arthroplasty over a long-term period of 11 years through both radiological and histomorphometric evaluations.

The primary limitation of our study is that it is based on a single case. Future studies with a larger number of patients and a focus on long-term outcomes are needed to provide further insights and guide our colleagues in clinical practice.

A limitation of this report is the lack of Von Kossa staining, which is considered the gold standard for demonstrating calcium salt deposition. Our specimen required decalcification for sectioning and immunohistochemical assessment, precluding meaningful Von Kossa analysis. We therefore relied on H&E and von Gieson stains to demonstrate maintained trabecular architecture and direct bone–implant contact, acknowledging that these methods provide morphological but not mineral-specific confirmation of osseointegration.

## 7 Conclusion

This study demonstrates that HemiCAP® surface arthroplasty can achieve long-term survival of up to 11 years. No cases of HemiCAP® hip surface arthroplasty with such long-term survival have been reported in the literature. Our case is the first to evaluate the osseointegration of the HemiCAP® prosthesis not only clinically and radiographically but also through histopathologic analysis of the prosthesis and its surrounding tissue.

## Author contribution

The original idea was created by Onur Bilge. The methodology was validated Mustafa Cihat Avunduk and Ulku Kerimoglu. The data were collected by [Muhammed Furkan Kucuksen] and curated by [Onur Bilge]. The original draft was written by Onur Bilge and Muhammed Furkan Kucuksen. All authors made substantial contributions to the study's conception and design, and participated in the critical revision, editing, and final approval of the manuscript.

## Informed consent statement

Informed consent was obtained from the participant included in this study.

## Consent

The patient was informed that data from the case would be sub mitted for publication, and gave his written and signed consent for all surgeries and gave permission for publication of the data from this case, including photographs.

## Ethical approval

This study was approved by the Ethics Committee of [Konya Necmettin Erbakan University Meram Faculty of Medicine] (Approval No: [2024-5391]).

## Guarantor

Onur Bilge MD Prof.

## Research registration number

Not applicable.

## Data collection

Clinical and follow-up data were collected retrospectively from the patient's medical records with written informed consent for publication.

## Funding

This research did not receive any specific grant from funding agencies in the public, commercial, or not-for-profit sectors. All authors have no relevant conflicts of interest to declare.

## Conflict of interest statement

The authors declared no potential conflicts of interest with respect to the research, authorship, and/or publication of this article.

## References

[1] Bilge O., Doral M.N., Yel M., Karalezli N., Miniaci A. (2015). Treatment of osteonecrosis of the femoral head with focal anatomic-resurfacing implantation (HemiCAP): preliminary results of an alternative option. J. Orthop. Surg. Res..

[2] George G., Lane J.M. (2022). Osteonecrosis of the femoral head. JAAOS Glob Res Rev..

[3] Lea M.A., Barkatali B., Porter M.L., Board T.N. (2014). Osteochondral lesion of the hip treated with partial femoral head resurfacing: case report and six-year follow-up. Hip Int..

[4] Ricciardi B.F., Nocon A.A., Jerabek S.A., Wilner G., Kaplowitz E., Goldring S.R. (2016). Histopathological characterization of corrosion product-associated adverse local tissue reaction in hip implants: a study of 285 cases. BMC Clin. Pathol..

[5] Amstutz H.C., Esposito C., Campbell P. (2010). Long-term preservation of femoral bone following hemiresurfacing. Hip Int..

[6] Coathup M., Blunn G., Flynn N., Williams C., Thomas N. (2001). A comparison of bone remodelling around hydroxyapatite-coated, porous-coated and grit-blasted hip replacements retrieved at post-mortem. J. Bone Joint Surg. (Br.).

[7] Kerwan A., Al-Jabir A., Mathew G., Sohrabi C., Rashid R., Franchi T., Nicola M., Agha M., Agha R.A. (2025). Revised Surgical CAse REport (SCARE) guideline: an update for the age of artificial intelligence. Prem. J. Sci..

[8] Agha R.A., Mathew G., Rashid R., Kerwan A., Al-Jabir A., Sohrabi C., Franchi T., Nicola M., Agha M., PROCESS Group (2025). Revised Preferred Reporting Of Case Series in Surgery (PROCESS) guideline: updated and revised 2025. Premier Journal of Science.

[9] Bilge O., Doral M.N., Miniaci A. (2015). Focal anatomic resurfacing implantation for bilateral humeral and femoral heads’ avascular necrosis in a patient with Hodgkin’s lymphoma and literature review. Int. J. Surg. Case Rep..

[10] Yuan Z., Zhang W., Meng X., Zhang J., Tenglong T., Zhao Y. (2020).

[11] Amstutz H.C., Ebramzadeh E., Sarkany A., Le Duff M., Rude R. (2004).

[12] Doria C., De Santis V., Falcone G., Proietti L., De Santis E. (2003). Osseointegration in hip prostheses: experimental study in sheep. Int. Orthop..

[13] Ko Y.S., Ha J.H., Park J.W., Lee Y.K., Kim T.Y., Koo K.H. (2023). Updating osteonecrosis of the femoral head. Hip Pelvis.

